# LOXL2 catalytically inactive mutants mediate epithelial-to-mesenchymal transition

**DOI:** 10.1242/bio.20146841

**Published:** 2014-01-03

**Authors:** Eva P. Cuevas, Gema Moreno-Bueno, Giacomo Canesin, Vanesa Santos, Francisco Portillo, Amparo Cano

**Affiliations:** 1 Departamento de Bioquímica, Universidad Autónoma de Madrid (UAM), Instituto de Investigaciones Biomédicas “Alberto Sols” (CSIC-UAM), IdiPAZ, 28029 Madrid, Spain; 2 Fundación MD Anderson International, 28033 Madrid, Spain; * Present address: Department of Experimental Pathology, Clinical Research Center, Jan Waldenströms gata 35, 20502 Malmö, Sweden

**Keywords:** LOXL2, EMT, Lysyl oxidase, FAK, Src

## Abstract

Lysyl-oxidase-like 2 (LOXL2) is a member of the lysyl oxidase family that catalyzes the cross-linking of collagens or elastins in the extracellular matrix, thus regulating the tensile strength of tissues. However, many reports have suggested different intracellular roles for LOXL2, including the ability to regulate gene transcription and tumor progression. We previously reported that LOXL2 mediates epithelial-to-mesenchymal transition (EMT) by Snail1-dependent and independent mechanisms, related to E-cadherin silencing and downregulation of epidermal differentiation and cell polarity components, respectively. Whether or not the catalytic activity of LOXL2 is required to induce/sustain EMT is actually unknown. Here we show that LOXL2 catalytic inactive mutants collaborate with Snail1 in *E-cadherin* gene repression to trigger EMT and, in addition, promote FAK/Src pathway activation to support EMT. These findings reveal a non-conventional role of LOXL2 on regulating epithelial cell plasticity.

## Introduction

LOXL2 is a member of the lysyl oxidase (LOX) family, constituted by five members, the prototypical LOX and four related members, lysyl oxidase-like (LOXL1–4). All LOX members share a highly conserved carboxyl (C)-terminus domain that contains a copper-binding motif, a lysyl-tyrosyl-quinone (LTQ) group, both essential for the catalytic activity (Lopez and Greenaway, 2011), and a cytokine receptor-like (CRL) domain of unknown function (Csiszar, 2001; Jourdan-Le Saux et al., 1999; Molnar et al., 2003). The catalytic domain is required for the oxidative deamination of peptidyl-lysine residues in substrates to generate reactive aldehyde groups that initiate covalent inter and intra-molecular crosslinking (Lucero and Kagan, 2006). In contrast to the highly conserved C-terminal domains, the amino (N)-terminal region of the LOX family members show sequence divergence. LOXL2–4 proteins contain four scavenger receptor cysteine-rich (SRCR) domains in their N-terminal region (Saito et al., 1997). The functional role of the SRCR domains in LOXL2–4 proteins has not yet been fully characterized, although they could be involved in protein–protein interactions and ligand binding in both soluble proteins and membrane-bound protein receptors (Hohenester et al., 1999; Martínez et al., 2011).

Although the role of LOXL2 in the maturation of extracellular matrix (ECM) is well established, there is increasing evidence involving LOXL2 in other physiological and pathological processes. Of remarkably interest is the implication of LOXL2 in the regulation of epithelial-to-mesenchymal transition (EMT) and tumor progression (Cano et al., 2012).

EMT, an essential process in development, is presently considered a key event in tumor progression, facilitating the dissemination of tumor cells and the acquisition of migratory properties that provides tumor cells the ability to invade the adjacent tissues (Thiery et al., 2009). A critical step in EMT is the downregulation of *E-cadherin* gene (*CDH1*) expression (Cano et al., 2000; Batlle et al., 2000). Several transcription factors have been described as EMT inducers and *CDH1* repressors (presently called EMT-TFs), including members of the Snail, bHLH and ZEB families (Peinado et al., 2007; Polyak and Weinberg, 2009; Nieto and Cano, 2012). LOXL2 plays a dual role in EMT induction. First, it interacts with and stabilizes Snail1, promoting *CDH1* silencing and, thus, inducing EMT (Peinado et al., 2005a). Second, LOXL2 helps to maintain the mesenchymal phenotype due, at least in part, to downregulation of cell polarity and epidermal differentiation genes in a Snail1-independent fashion (Peinado et al., 2008; Moreno-Bueno et al., 2011), associated to activation of FAK kinase (Moreno-Bueno et al., 2011).

Recently, it has been shown that blocking stromal LOXL2 by specific inhibitory monoclonal antibodies abrogates the formation of the pathologic microenvironment in cancer and fibrotic diseases (Barry-Hamilton et al., 2010) and the metastatic potential of breast carcinoma cells (Barker et al., 2011). These data suggest that the catalytic activity of the extracellular LOXL2 is required for the development of a tumor pathologic microenvironment. A histone deaminase function has also been recently reported for intracellular LOXL2 (linked to heterochromatin (Herranz et al., 2012; Millanes-Romero et al., 2013)), but the biological implication of this new LOXL2 enzymatic function is not yet fully understood. Enzymes such heparanase and DNA metiltransferase 1 have been found to induce biological responses by mechanisms independent of their catalytic activity (Fux et al., 2009; Espada et al., 2011). In line with these later findings, two recent reports indicate that LOXL2 catalytic activity is required neither to inhibit keratinocyte differentiation nor to repress *Claudin-1* and *Lgl2* promoter activity (Lugassy et al., 2012; Moreno-Bueno et al., 2011). We have further explored the implication of LOXL2 catalytic activity for EMT induction. Here we provide evidence that catalytically inactive LOXL2 mutants, one of them unable to be secreted, induce and sustain a full EMT process, indicating that intracellular LOXL2 drives EMT independent of its enzymatic activity.

## Results

### LOXL2 catalytically inactive mutants

**Fig. 1.**
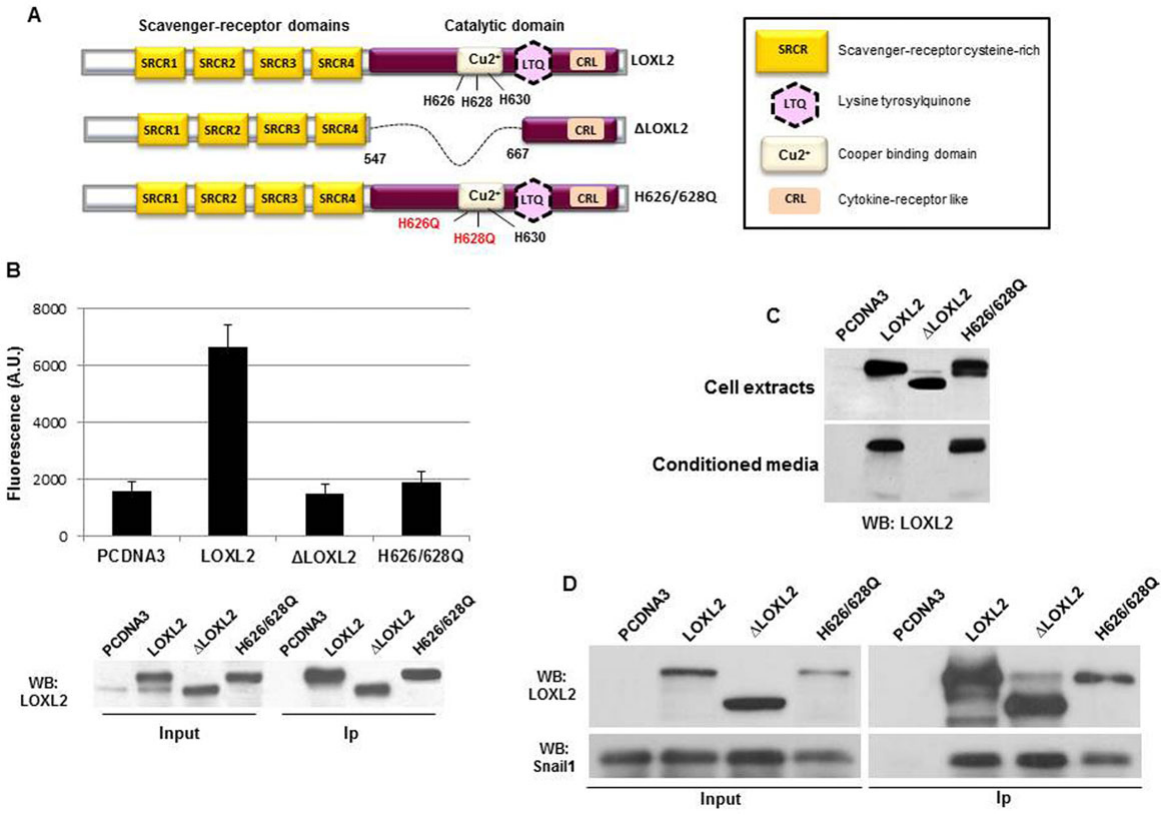
Characterization of LOXL2 catalytically inactive mutants. (A) Schematic representation of wild-type LOXL2 (upper panel), deletion mutant ΔLOXL2 (middle) and double point mutant LOXL2-H626Q/H628Q (H626/628Q) (lower panel). (B) Enzymatic activity of wild-type LOXL2, ΔLOXL2, and H626/628Q mutants (upper panel). LOXL2 variants were immunoprecipitated from whole cell extracts of HEK293T, transiently transfected with the indicated LOXL2 forms, with anti-Flag M2 affinity gel (LOXL2 and ΔLOXL2) or anti-HA antibodies (H626/628Q) and activity assayed as described in Materials and Methods. Activity detected after transfection of a void pcDNA3 vector is included as blank data. Data are the mean of two independent experiments. Western blots showing the levels of LOXL2 proteins in the whole cell extract (input) and in the immunoprecipitated (Ip) fractions are showed in the lower panel. (C) Western blot analyses of intracellular (upper panel) and extracellular LOXL2 (lower panel) proteins from HEK293T cells transiently transfected with the indicated LOXL2 variants. (D) Co-immunoprecipitation assay. Whole cell extracts from HEK293T cells transiently co-transfected with Snail1-HA and either pcDNA3, LOXL2-Flag, ΔLOXL2-Flag or LOXL2-H626/628Q-HA were immunoprecipitated with anti-Flag M2 affinity gel (LOXL2 and ΔLOXL2) or anti-LOXL2 antibody (H626/628Q) and analysed by Western blot with anti-LOXL2 or anti-HA to detect the association of the indicated LOXL2 variants and Snail1 (right panel). Detection of the corresponding Snail1 and LOXL2 proteins in the input fractions is shown in the left panel.

We previously identified LOXL2 as a Snail1 interacting partner contributing to the Snail1-mediated silencing of E-cadherin and as an EMT driver (Peinado et al., 2005a). In order to clarify the implication of LOXL2 enzymatic activity in such processes we created two LOXL2 mutants affecting the conserved catalytic domain (Fig. 1A). One of the mutants (ΔLOXL2) carries a 120 amino acids deletion (from 547 to 667) that eliminates the Cu^2+^-binding motif, the Lys655 residue required for formation of the LTQ co-factor (Lopez and Greenaway, 2011) and the Asn646 residue involved in N-glycosylation and required for LOXL2 secretion (Xu et al., 2013). The second mutant contains two point mutations in the conserved Cu^2+^-binding domain of LOXL2 affecting His626 and His628 (H626/628Q). These amino acids were selected based on previous reports showing the role of equivalent positions in other members of the Lox family for enzyme activity (Lopez and Greenaway, 2011; Lugassy et al., 2012; Herranz et al., 2012; Xu et al., 2013). Enzyme activity was determined in immunoprecipitated LOXL2 proteins following ectopic expression in HEK293T cells by coupled fluorimetric assays for H_2_O_2_ production. In equivalent amounts of immunoprecipitated LOXL2 forms (Fig. 1B, bottom) the activity of ΔLOXL2 and H626/628Q mutants was similar to that of the blank vector control (Fig. 1B, upper), in concordance with the activity level previously reported in other catalytically inactive LOXL2 mutants (Lugassy et al., 2012; Herranz et al., 2012). The ΔLOXL2 and H626/628Q mutants were expressed at similar levels than LOXL2 in HEK293T cells as detected in whole cell extracts (Fig. 1C, upper); however, ΔLOXL2 mutant was not secreted to the conditioned medium in contrast to wild-type LOXL2 and the H626/628Q mutant (Fig. 1C, bottom). To ascertain whether LOXL2 variants retained the ability to interact with Snail1 co-immunoprecipitation experiments were performed in HEK293T cells transiently transfected with tagged versions of Snail1, LOXL2, ΔLOXL2 and H626/628Q. Co-immunoprecipitation of Snail1 by LOXL2, ΔLOXL2 and H626/628Q (Fig. 1D) indicates that LOXL2 mutants are still able to interact with Snail1.

### LOXL2 inactive mutants bind to the E-boxes of the CDH1 promoter and repress E-cadherin expression

**Fig. 2.**
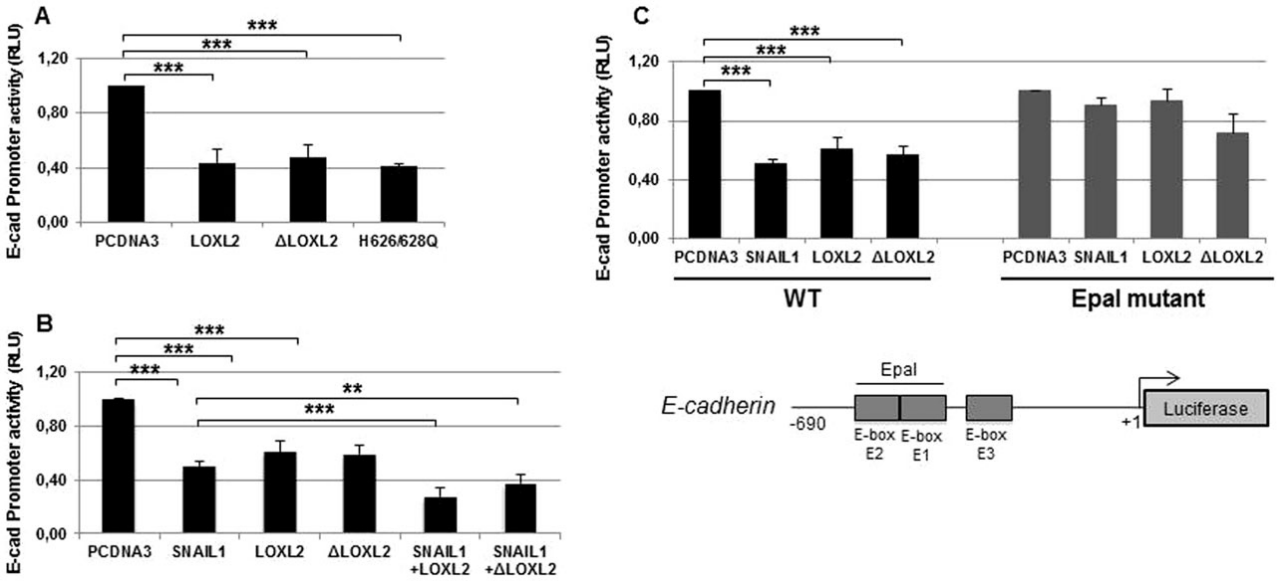
E-cadherin is transcriptionally repressed by LOXL2. (A) The activity of the *E-cadherin* promoter in HEK293T cells was measured in the presence of the indicated LOXL2 variants (50 ng). (B) *E-cadherin* promoter activity in HEK293T cells was measured in the presence of the indicated LOXL2 forms (50 ng) and in the absence or presence of Snail1 (50 ng). The effect of Snail1 (50 ng) in the absence of LOXL2 and ΔLOXL2 was also tested. (C) Activity of the wild-type (left) and mutant *E-cadherin* promoter (Epal mutant) (right) in HEK293T cells was measured in the presence the indicated factors (50 ng). Schematic representation of the proximal mouse *E-cadherin* promoter is shown at the bottom. In all cases, the activity was determined as relative luciferase units (RLU) and normalized to the activity detected in the presence of control pcDNA3 vector. Results represent the mean ± s.e.m. of at least three independent experiments performed in triplicate samples (*p<0.05, **p<0.005, ***p<0.001).

We previously identify LOXL2 as a Snail1-interacting partner contributing to the Snail1-mediated silencing of *E-cadherin* (Peinado et al., 2005a). Promoter assays indicated that the activity of the *E-cadherin* promoter was downregulated by LOXL2 independently of its catalytic domain since both the ΔLOXL2 and H626/628Q mutants showed the same repressor potency as wild-type LOXL2 (Fig. 2A). In addition, LOXL2 inactive mutants conserved its ability to cooperate with Snail1 to repress the *E-cadherin* promoter (Fig. 2B). It is well established that transcription factors repressing *E-cadherin* expression, like Snail1, bind to the E-pal element present in the mouse *Cdh1* proximal promoter and that mutations in this element preclude *E-cadherin* repression (Bolós et al., 2003; Cano et al., 2000). To analyze if the transcriptional repression exerted by wild type and inactive LOXL2 mutants required the E-pal element, promoter assays were performed with a reporter gene carrying E-pal mutations in the presence of LOXL2 or the ΔLOXL2 mutant. As a control we also included Snail1 in the assay. Fig. 2C shows that repression of the *E-cadherin* promoter by Snail1 and LOXL2 variants is abolished in the E-pal mutant, indicating that repression in all cases depend on the proximal E-boxes.

**Fig. 3.**
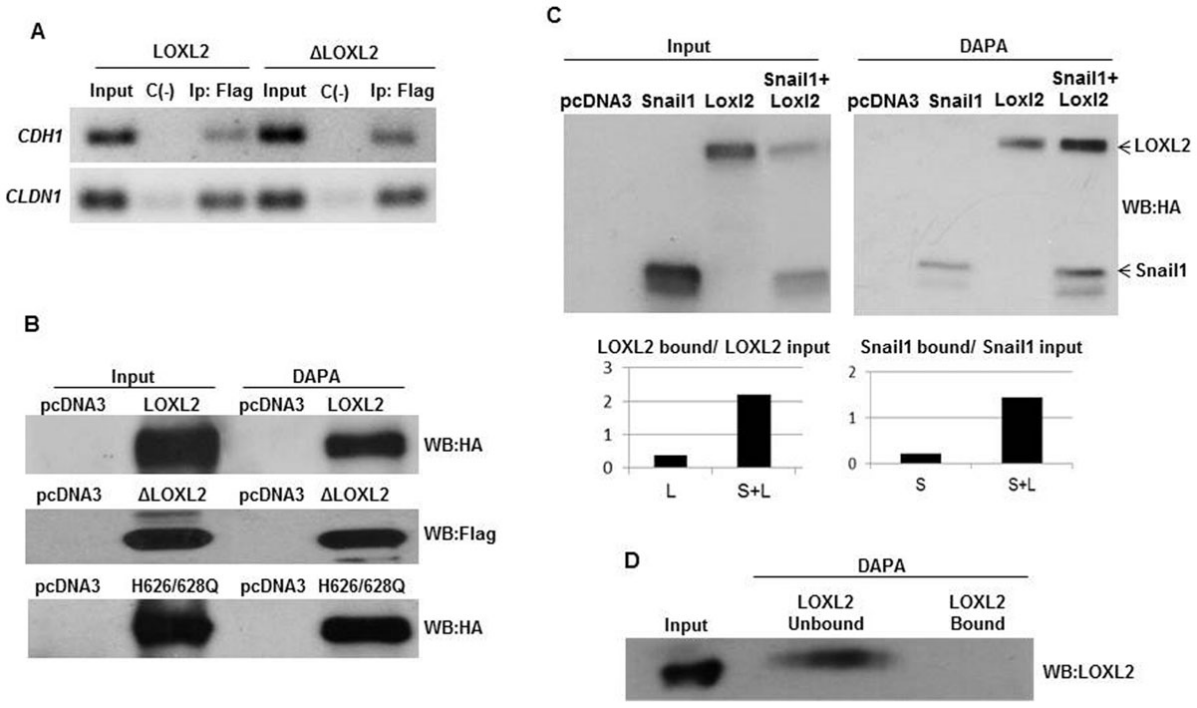
LOXL2 binds to the endogenous E-cadherin promoter. (A) Chromatin immunoprecipitation assays were performed in HEK293T cells transiently transfected with LOXL2-Flag or ΔLOXL2-Flag. For detection of interaction between the tagged factors and the endogenous *CDH1* or *CLDN1* promoters, an M2 affinity gel or unspecific rabbit IgG were used. A 218 bp fragment of *CDH*1 promoter (−180/+38) or 80 bp fragment of *CLDN1* promoter (+146/+245) was amplified using the primers and amplification conditions described in Materials and Methods. (B) The binding of LOXL2-HA, ΔLOXL2-Flag or H626/628Q-HA to the biotinylated E-pal element of mouse *E-cadherin* promoter was performed by DAPA assay and analysed by Western blot as described in Materials and Methods. Detection of the corresponding LOXL2 proteins in the input fractions is shown in the left lanes. In panels A and B, cells transfected with the empty pcDNA3 vector were used as control. (C) The binding of Snail1, LOXL2-HA and Snail1+LOXL2 to the E-pal element was analysed by DAPA assay. Detection of the corresponding Snail1 or LOXL2 proteins in the input fractions is shown in the left panel. Quantifications of the proteins bound to the probe in each case were normalized to the input fraction (lower panel). (D) The binding of purified (>90%) LOXL2 protein (300 ng) to the E-pal element of *CDH1* promoter was analysed by DAPA assay. Detection of the corresponding LOXL2 protein in the input fraction is shown in the left lane.

We next analyzed the binding of LOXL2 and ΔLOXL2 to the *E-cadherin* promoter by chromatin immunoprecipitation (ChIP) and DNA-affinity precipitation (DAPA) assays. ChIP assays showed that LOXL2 and ΔLOXL2 were able to interact with the *CDH1* promoter (Fig. 3A) in a 218 bp region that contains the E-pal element. Importantly, ΔLOXL2 was also able to interact with the human *CLAUDIN*-1 (*CLDN1*) promoter (Fig. 3A) in a region that includes the E-boxes. We confirmed the binding of wild-type LOXL2 and ΔLOXL2 and H626/628Q variants specifically to the E-pal element of the *E-cadherin* promoter by DAPA assays (Fig. 3B). Interestingly, co-expression of LOXL2 and Snail1 reinforces the binding of both molecules to the E-pal element (Fig. 3C) and similar effect was observed after co-expression of ΔLOXL2 and H626/628Q mutants (supplementary material Fig. S1).

We next asked whether the binding of LOXL2 to the E-pal element of the *E-cadherin* promoter was direct. DAPA analysis using purified (>90%) LOXL2 protein indicates that it required of accessories proteins (Fig. 3D).

All together, the data suggest that the binding of LOXL2 to E-boxes is indirect and that LOXL2 repression potency is unconnected to its catalytic activity.

**Fig. 4.**
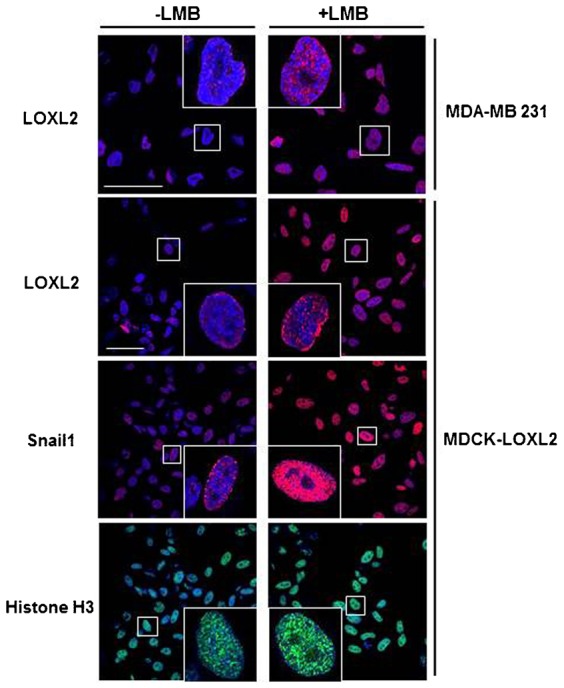
Translocation of LOXL2 into the nucleus. Accumulation of LOXL2 within the nucleus in MDA-MB231 or MDCK-LOXL2 cells was analyzed by confocal immunofluorescence in the absence or presence of Leptomycin B (LMB; 5 ng/ml, 16 h). Distribution of Snail1 and histone H3 were also included as control. Images show only the overlap between red (LOXL2 and Snail1) or green signals (Histone H3) and DAPI staining. Scale bars: 50 µm.

We have previously described that intracellular distribution of LOXL2 is mainly cytoplasmic and/or perinuclear (Peinado et al., 2005a) and that perinuclear staining is a poor prognosis marker in larynx squamous cell carcinomas (Peinado et al., 2008) and associated to metastasis of basal-like breast tumors (Moreno-Bueno et al., 2011). Nevertheless, the fact that LOXL2 binds to the *E-cadherin* promoter also strongly suggests a nuclear localization for LOXL2, as also was previously suggested by other group (Herranz et al., 2012). To check if LOXL2 can translocate into the nucleus, we analyzed the distribution of endogenous (MDA-MB-231) or overexpressed LOXL2 (MDCK-LOXL2). In basal conditions, some LOXL2 staining is observed within the nucleus (Fig. 4, left). Treatment of cells with Leptomycin B, a nuclear export inhibitor, led to a nuclear accumulation of LOXL2 (Fig. 4, right). These data suggest that, at least a fraction of LOXL2 can move into the nucleus, thus supporting the repressive action of LOXL2 on epithelial gene promoters and its indirect binding to the *E-cadherin* promoter.

### The enzymatic activity of LOXL2 is not required for the induction of EMT

**Fig. 5.**
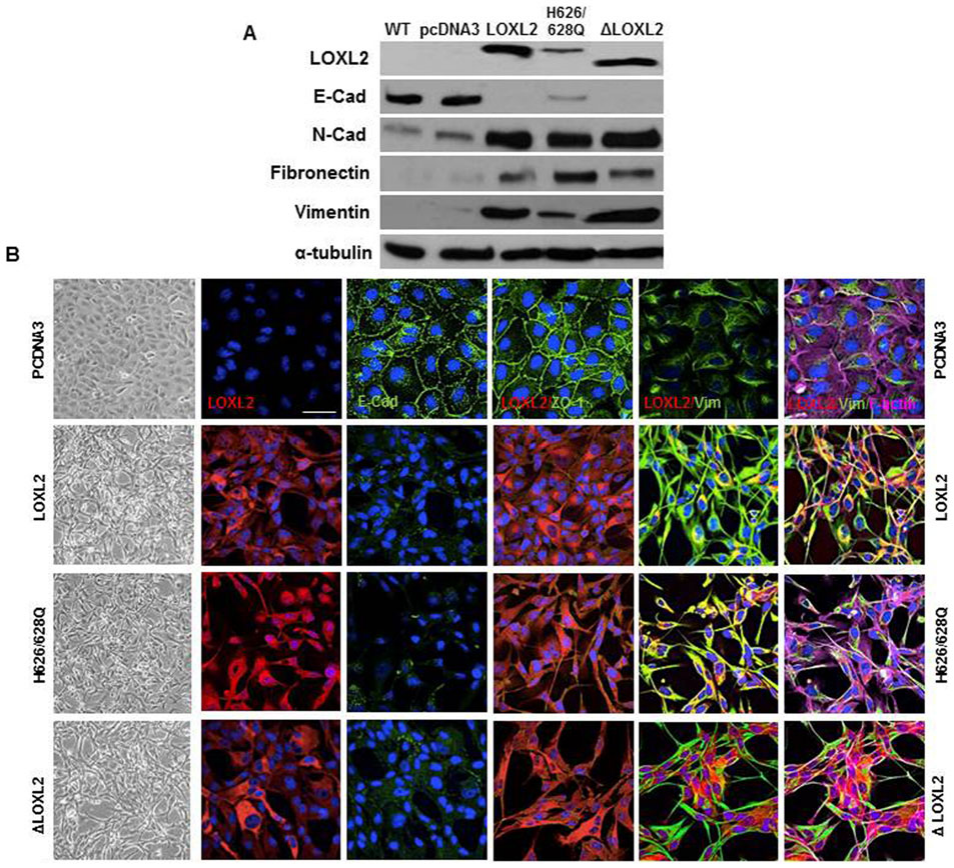
The stable expression of LOXL2 mutants in MDCK cells induces EMT. Characterization of MDCK transfectants obtained after stable expression of wild-type LOXL2, and the LOXLi mutants ΔLOXL2 or H626/628Q. (A) Western blot analyses were performed on whole cell extracts for the expression of ectopically expressed LOXL2 variants, E-cadherin, N-cadherin, fibronectin and vimentin. α-tubulin was used as a loading control. (B) Confocal immunofluorescence images for ectopically expressed LOXL2 variants (red), E-cadherin/ZO-1/vimentin (green) and F-actin (magenta) in the indicated clones. Phase contrast images of the indicated cells are shown at the left panels. Scale bar: 50 µm.

**Fig. 6.**
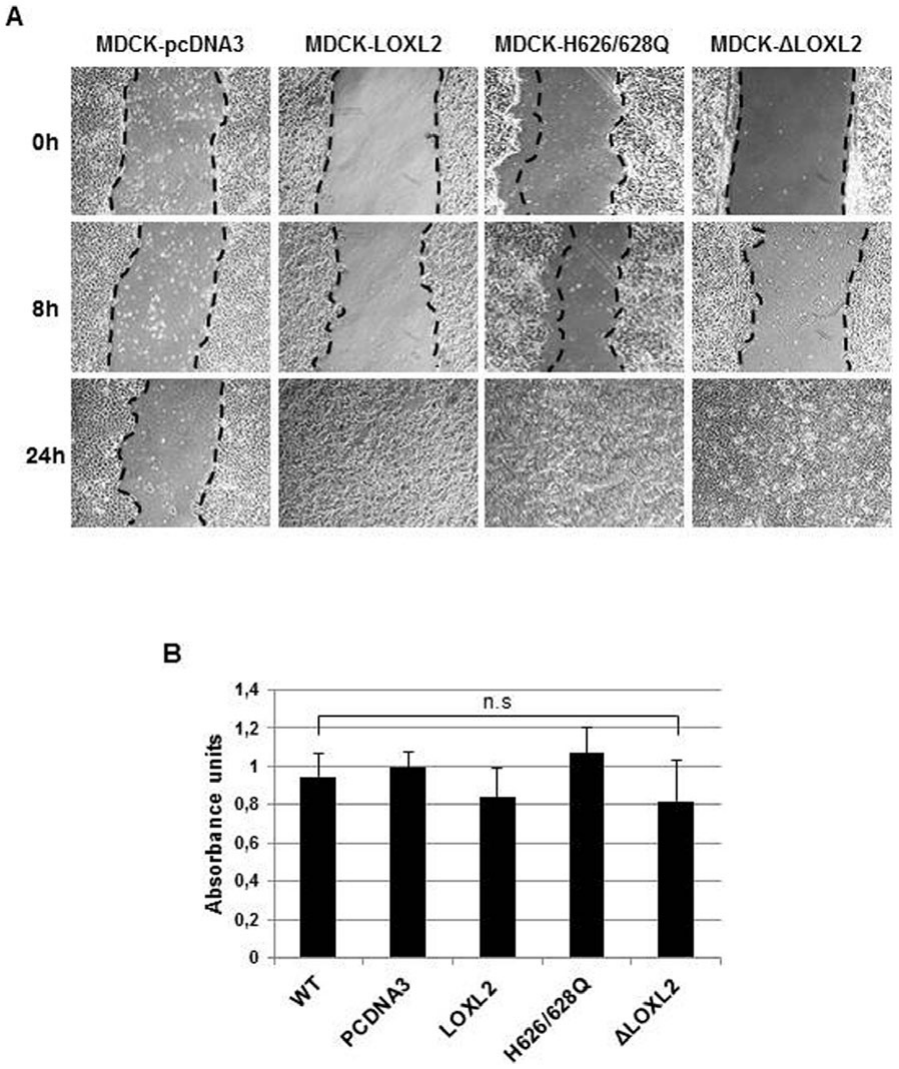
Effect of stable expression of LOXL2 variants in cell motility. (A) Cell motility of the indicated MDCK clones was analyzed by wound healing assay. Images were taken at 0 h, 8 h and 24 h after culture scratch. (B) Proliferation of the MDCK clones was measured by the MTT reduction assay at 48 h of growth. Results represent the mean of two experiments performed in triplicate samples; n.s.  =  not significant.

The data presented so far showed that LOXL2 enzymatic activity is dispensable for E-cadherin repression, and a similar situation was previously reported for Claudin 1 and Lgl2 genes (Moreno-Bueno et al., 2011). We previously reported that stable expression of LOXL2 in MDCK cells induces the loss of E-cadherin and a complete EMT (Peinado et al., 2005a). To further characterize the implication of LOXL2 enzymatic activity in EMT induction, we examined the phenotype of MDCK cells stably expressing wild-type LOXL2 or catalytically inactive (ΔLOXL2 and H626/628Q) mutants. As a control, we analyzed MDCK cells transfected with the empty vector (pcDNA3). Concordant with our previous reports stable expression of LOXL2 in MDCK cells induced a conversion to a fibroblastic/spindle phenotype compared to control cells that exhibit an unaltered epithelial phenotype (Fig. 5B, upper two left panels). Remarkably, the expression of the ΔLOXL2 and H626/628Q mutants induced the same phenotypic effect than wild-type LOXL2 (Fig. 5B, bottom two left panels). Analyses of epithelial and mesenchymal markers by both Western blot (Fig. 5A) and immunofluorescence (Fig. 5B) showed that stable expression of wild type or LOXL2 mutants provoke a complete loss of E-cadherin and ZO-1 with the concomitant increase in mesenchymal markers (N-cadherin, vimentin and fibronectin) and the F-actin cytoskeleton exhibiting a typical organization of mesenchymal cells. It should be noted that the levels of ectopically expressed LOXL2 (wt and inactive mutants) in MDCK cells are similar or even lower to the endogenous LOXL2 levels detected in several human breast basal like cells (supplementary material Fig. S2) in which LOXL2 plays a functional role in maintenance of the mesenchymal phenotype and metastatic behavior (Moreno-Bueno et al., 2011). Together, these data indicate that the ability of LOXL2 to induce a complete EMT is unlinked to its catalytic activity. Additionally, we examined the effect of the different LOXL2 forms on the motile phenotype by wound healing assays. As shown in Fig. 6A expression of wild type and catalytically inactive LOXL2 mutants resulted in a marked increase in cell motility, which was not due to increased proliferation rate, as confirmed by MTT assays (Fig. 6B).

### The enzymatic activity of LOXL2 is not required for activation of FAK/Src pathway

**Fig. 7.**
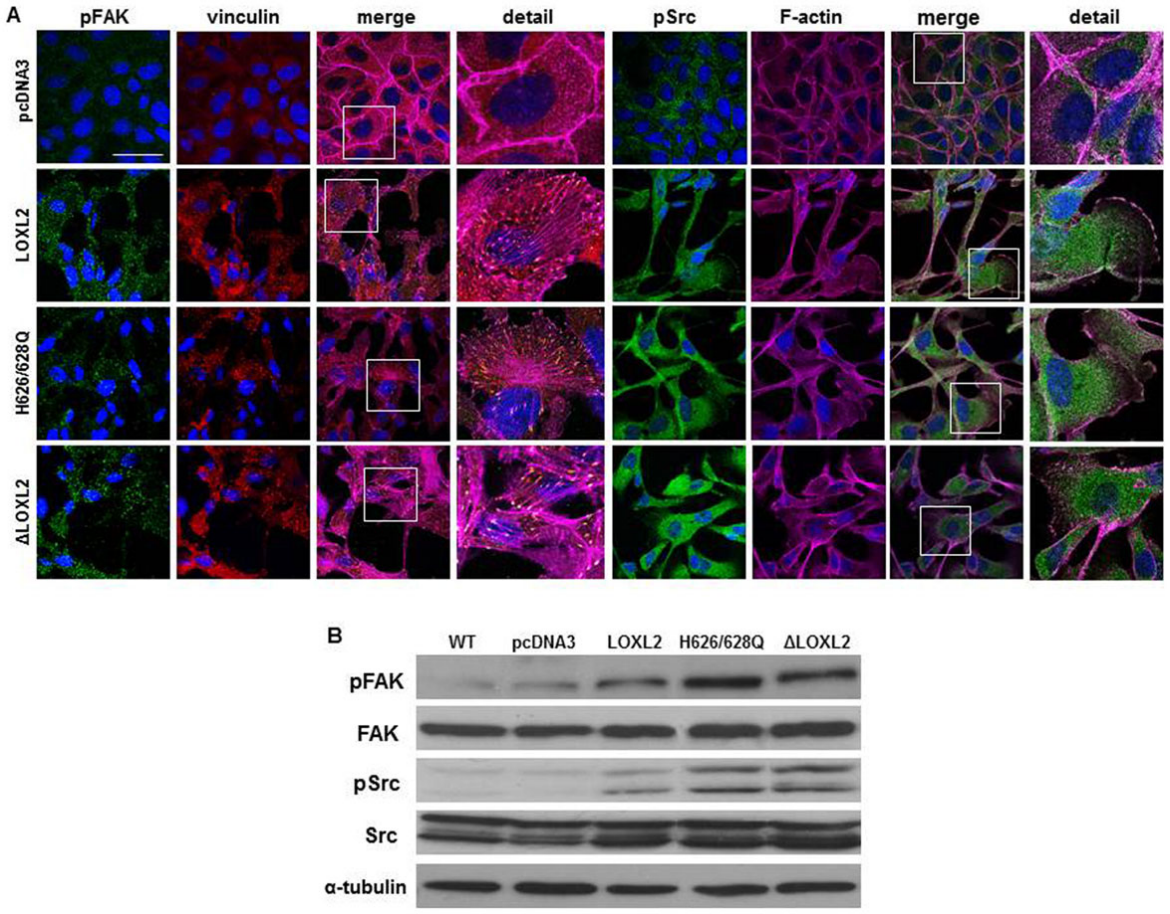
LOXL2 catalytically inactive mutants promote FAK/Src activation. (A) Confocal immunofluorescence images for p-FAK^Y397^ and p-Src (green), vinculin (red) and F-actin (magenta) in the indicated MDCK clones. Merge and amplified (detail) images are shown as indicated for pFAK/vinculin (middle left columns) and pSrc/F-actin (right columns). Scale bar: 50 µm. (B) Western blot analyses were performed on whole cell extracts for p-FAK and p-Src and total expression levels in the indicated cells. α-tubulin was used as a loading control.

An earlier report showed that secreted LOXL2 positively modulate the FAK/Src signaling pathways in gastric carcinoma cells (Peng et al., 2009). Additionally, in a recent report we have described that LOXL2 contributes in a Snail1-independent fashion to FAK signaling pathway activation in basal-like carcinoma cells (Moreno-Bueno et al., 2011), suggesting that FAK activation might contribute to LOXL2 mediated downregulation of cell polarity components and maintenance of the mesenchymal phenotype in those cells. Therefore we next decided to analyze whether LOXL2 catalytic activity is required for FAK activation. We studied the influence of LOXL2 constructs on FAK activation and focal adhesion organization by confocal immunofluorescence of p-FAK, p-Src, vinculin and F-actin. MDCK-pcDNA3 control cells displayed low and diffuse p-FAK and vinculin staining and lack organized focal contacts; instead, F-actin is organized in cortical bundles typical of epithelial cells (Fig. 7A, upper panels). By contrast, MDCK cells expressing wild-type or mutant LOXL2 showed abundant focal contacts with clear co-localization between p-FAK and vinculin in the anchorage zones of the stress fibers as well as between p-Src and F-actin stress fibers (Fig. 7A, middle and lower panels) suggestive of FAK/Src signaling pathway activation. Activation of FAK and Src by either wild type or LOXL2 mutants was confirmed by Western blot analysis (Fig. 7B). Taken together, these results indicate that FAK activation is independent of LOXL2 catalytic activity and appears to be mediated by intracellular LOXL2.

## Discussion

We provide here data indicating a non-enzymatic role of LOXL2 in the induction and maintenance of the EMT process. To dissect the mechanism underlying LOXL2 effect on EMT we created two catalytically inactive mutants (LOXL2_i_) and explored its impact on different EMT-related parameters. Both wild-type and LOXL2_i_ enzymes exhibit the same competency in binding and silencing specifically the *E-cadherin* promoter, which in turn propels the EMT process. The phenotype of the EMT driven by LOXL2_i_ is indistinguishable of that induced by the wild-type allele. Our results also suggest that the H3K4me3 deaminase activity of LOXL2 proposed recently (Herranz et al., 2012) is dispensable for E-cadherin repression and EMT induction.

Concerning to the process mediating repression of *CDH1* gene expression by LOXL2_i_, we favored the hypothesis that LOXL2 could act on Snail1 through two overlapped and concurrent mechanism. One of the mechanisms is dependent of the catalytic activity of the enzyme that counteracts the action of GSKβ3 that leads to Snail1 stabilization as proposed previously (Peinado et al., 2005b). The other one is independent of LOXL2 catalytic activity and would be a secondary effect of the interaction between LOXL2 and Snail1. It is tempting to speculate that LOXL2_i_-Snail1 interaction prompts Snail1 to adopt a more active conformation with enhanced DNA binding ability as suggested by the DAPA analysis. This is a difficult to prove hypothesis but, in support of this explanation, we report here that LOXL2_i_ have an additive effect on Snail1-dependent *CDH1* silencing (Fig. 2) and that co-expression of LOXL2_i_ and Snail1 increase the amount of Snail1 bound to the E-pal element of *CDH1* promoter (Fig. 3B; supplementary material Fig. S1).

Wild-type LOXL2 contributes positively to activation of the FAK signaling pathway. FAK is a cytoplasmic non-receptor tyrosine kinase that is activated at sites of integrin-mediated cell adhesions and by growth factor receptors impinging on a number of biological processes involved in neoplastic transformation, invasion and metastases (Schaller, 2010). Since FAK responds to extracellular stimuli, including signals from the extracellular matrix (ECM) (Schaller, 2010), it is not surprising that wild-type LOXL2, an ECM remodeling enzyme, activates the FAK signaling pathway as has been reported in both gastric and basal-like carcinoma cells (Peng et al., 2009; Moreno-Bueno et al., 2011). We have found, however, that LOXL2_i_ mutants are as competent as the wild-type form to activate FAK, even in the absence of secretion, as in the case of the ΔLOXL2 variant. In this latter case, we have to postulate that activation of FAK by LOXL2_i_ has to result from indirect intracellular mechanisms. Recently, it has been reported that loss of E-cadherin in cell models of incipient stages of squamous cell carcinoma drives the upregulation of FAK mRNA and protein levels as well as FAK/Src activities (Alt-Holland et al., 2011). Therefore, it is plausible to assume that the downregulation of E-cadherin detected in cells expressing LOXL2_i_ mutants can account for the observed FAK activation. Since the expression levels of ectopic LOXL2 and LOXL2i mutants are similar, or even lower, to the endogenous LOXL2 levels present in several mesenchymal basal carcinoma cells, it is unlikely that the observed effects in MDCK cells are due to overexpression of the proteins. Nevertheless, some indirect effects due to elevated levels of the ectopic wt and ΔLOXL2 proteins cannot be fully discarded at present.

In conclusion, the results presented here reveal that LOXL2 conserves biological functions on regulating EMT and epithelial cell plasticity beyond its enzymatic activity. These functions are achieved, at least, through the silencing of *E-cadherin* gene expression and activation of the FAK/Src pathways.

## Materials and Methods

### Cell culture and transfection

HEK293T and MDCK-II cell lines were obtained from the American Type Culture Collection and grown in DMEM medium (Gibco), supplemented with 10% fetal bovine serum, 10 mmol/L glutamine (Life Technologies), 100 µg/mL ampicillin and 32 µg/mL gentamicin. All cell lines were grown at 37°C in a humidified 5% CO_2_ atmosphere. Stable transfectants were obtained from parental MDCK-II after transfection with pReceiver-hLOXL2-HA, pcDNA3-ΔLOXL2-Flag, pReceiver-hLOXL2-H626/628Q-HA or pcDNA3 empty vector, using Lipofectamine (Invitrogen). Cells were grown in the presence of G418 (400 µg/ml) for 3–4 weeks and individual clones isolated with cloning rings. At least 10 independent clones were isolated from each transfection. Three independent clones from each transfection were analyzed and results representative of one single clone are shown in the figures.

### Plasmid constructs

The human pcDNA3-LOXL2-Flag, mouse pcDNA3-LOXL2-Δ547-667-Flag (ΔLOXL2) and human pcDNA3-hSnail1-HA vectors have been previously described (Peinado et al., 2004; Peinado et al., 2005a; Moreno-Bueno et al., 2011). The human pReceiver-LOXL2-HA was purchased to GeneCopoeia (Source BioScience). LOXL2-H626/628Q-HA mutant was generated from pReceiver-LOXL2-HA by site-directed mutagenesis (Mutagenex Inc). The mouse *E-cadherin* promoter (−178 to +92) fused to the luciferase reporter gene in its wild-type and mutant Epal (mEpal) version, was previously described (Bolós et al., 2003).

### Promoter assays

Cotransfections were carried out in the presence of 50 ng of Snail1 and/or LOXL2 (wild type or mutants) cDNAs, 200 ng of the indicated promoter and 10 ng of pCMV-β-gal as control of transfection efficiency. The amount of total DNA was normalized with empty pcDNA3 vector (up to 100 ng). Luciferase and β-galactosidase activities were measured using the luciferase and β-Glo assay substrates (Promega) and normalized to the wild-type promoter activity detected in cells transfected with pcDNA3 empty vector.

### Cell extracts, Western blot and immunoprecipitation analyses

Cell extracts, immunoprecipitation conditions and Western blot analyses were performed as previously described (Moreno-Bueno et al., 2009; Peinado et al., 2004). The antibodies used are described in supplementary material Table S1.

### DNA affinity purification assay (DAPA)

DNA precipitations were carried out essentially as described previously (Hata et al., 2000). Briefly, cell extracts or purified (>90%) human LOXL2 (R&D Systems) were precleared with Dynabeads M-280 Streptavidin (Invitrogen) for 1 h, then incubated with 2 µg of biotinylated double-stranded oligonucleotides corresponding to the E-pal element of the mouse *E-cadherin* promoter (5′-GGCTGCCACCTGCAGGTGCGTCCC-3′), together with 2 µg of poly(dI-dC) for 16 h. DNA-bound proteins were collected with Dynabeads M-280 Streptavidin for 3 h and analyzed by Western blotting.

### Chromatin immunoprecipitation (ChIP) assay

ChIP assays were performed in HEK293T-cells transiently transfected with either LOXL2-Flag or ΔLOXL2-Flag, using formaldehyde before sonication, as described (Peinado et al., 2004; Sobrado et al., 2009). For detection of interaction between tagged factors with the endogenous *CDH1* or *CLDN1* promoters, anti-Flag M2 affinity gel (Sigma), or unspecific mouse IgG (Jackson ImmunoResearch Laboratories) and Protein G agarose beads (Sigma) were used. A 218 bp fragment of the human *CDH1* promoter (−180/+38) and 80 bp fragment of human *CLDN1* promoter (+146/+245), containing E-boxes (Martínez-Estrada et al., 2006; Sobrado et al., 2009), were amplified using the primers and amplification conditions previously described (Peiró et al., 2006; Sobrado et al., 2009).

### Immunofluorescence and confocal analyses

Immunofluorescence analysis was performed basically as described (Moreno-Bueno et al., 2009) on cells grown on coverslips and fixed on methanol or paraformaldehyde. Secondary antibodies, described in supplementary material Table S1, were anti-mouse, -rabbit or -goat Alexa 488/546, depending on the primary antibodies. Phalloidin-647 (Amersham) was used to stain F-actin. Confocal microscopy analyses were performed using a Leica Spectral TCS SP2, ×63 objective. Images were analyzed using ZEN (Zeiss) and Image J software.

### LOXL2 enzymatic activity

LOXL2 enzymatic activity was measured by coupling horseradish peroxidase (HRP) activity to LOXL2 and using the conversion of Amplex Red to resorufin, as described (Palamakumbura and Trackman, 2002). Intracellular LOXL2 variants transiently overexpressed in HEK293T cells were immunoprecipitated and suspended in reaction buffer (50 mM Sodium Borate pH 8.0, 1.2 M Urea, 10 mM CaCl_2_). Enzymatic reaction was started by adding 50 µl of substrate buffer (50 mM Sodium Borate pH 8.0, 40 µM Amplex Red, 2 U/ml Horseradish Peroxidase and 4 mM Benzylamine) and incubated at 37°C for 4 h. Samples were then centrifuged to separate agarose beads from the reaction mix. Fluorescence was measured on the supernatant in a Biotek Synergy HT microplate reader in endpoint mode with 530/25 nm excitation and 590/35 nm emission parameters.

### Migration assays

Wound healing assays were performed as described (Moreno-Bueno et al., 2009). Cultures were observed at timely intervals and phase-contrast pictures of the wounded area were taken using an inverted Zeiss Axiovert microscope.

### MTT reduction assay

The MTT assay was performed as previously described (Alley et al., 1988) with minor modifications. MTT stock solution in PBS buffer was added to the cell culture to obtain a final concentration of 0.5 mg/ml MTT. Absorbance was measured using Biotek Synergy HT microplate reader.

## Supplementary Material

Supplementary Material
