# Supplementary material for: LOXL2 catalytically inactive mutants mediate epithelial-to-mesenchymal transition
Source: Biol Open. 2014 Jan 3;3(2):129–37. doi: 10.1242/bio.20146841 (PMC3925316; doi:10.1242/bio.20146841)
Supplement: Supplementary Material [file supp_3_2_129__index.html]

LOXL2 catalytically inactive mutants mediate epithelial-to-mesenchymal transition — LOXL2 catalytically inactive mutants mediate epithelial-to-mesenchymal transition — Supplementary Material 

# LOXL2 catalytically inactive mutants mediate epithelial-to-mesenchymal transition

## bio.20146841 Supplementary Material

**Files in this Data Supplement:**

- Supplementary Material - Eva P. Cuevas et al. doi: 10.1242/bio.20146841
