# Supplementary material for: LOXL2 catalytically inactive mutants mediate epithelial-to-mesenchymal transition
Source: Biol Open. 2014 Jan 3;3(2):129–37. doi: 10.1242/bio.20146841 (PMC3925316; doi:10.1242/bio.20146841)
Supplement: Supplementary Material [file supp_bio.20146841_bio.20146841-s1.pdf]

Supplementary Material

Eva P. Cuevas et al. **doi: 10.1242/bio.20146841**

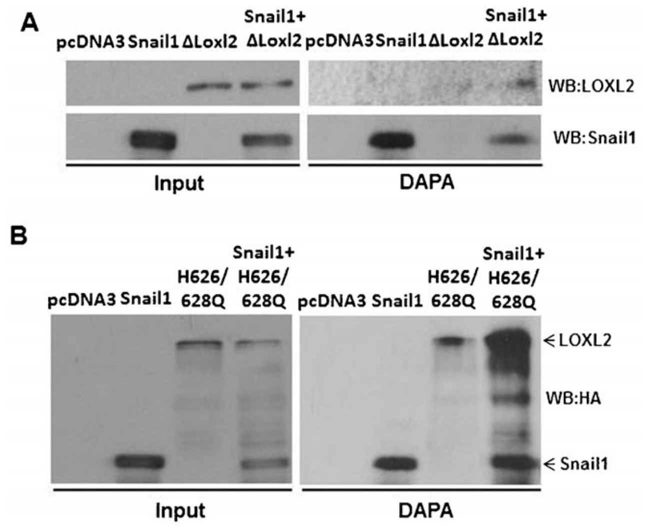

Fig. S1. Co-expression of Snail1 and LOXL2 mutants increases Snail1 binding to the E-pal element of the E-cadherin promoter. DAPA assays comparing the binding affinity to the E-pal probe of Snail1 and/or LOXL2 mutants. Increased Snail1 binding was observed in the presence of either ΔLOXL2 (A) or H626/628Q (B) mutant compared to the binding found when each factor was expressed individually in HEK293T cells.

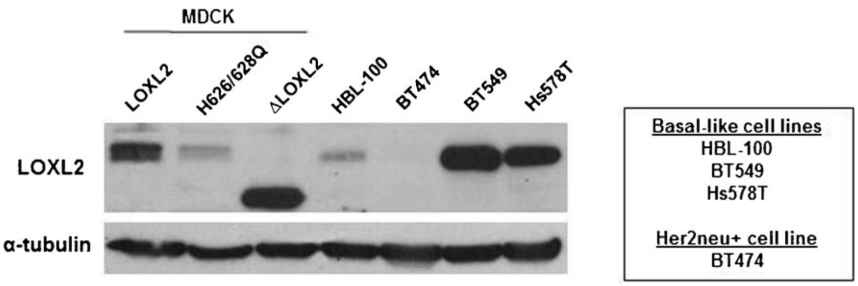

Fig. S2. Ectopic LOXL2 expression in MDCK cells compared to endogenous LOXL2 level in basal-like breast cancer cells. Western blot analyses of whole cell extracts from MDCK cells stably expressing wild-type LOXL2 and LOXL2i mutants, and the indicated human breast cells for LOXL2 using anti-LOXL2 antibodies. α-tubulin was used as a loading control. Among the basal-like cells, HBL-100 are non-tumorigenic and the rest are tumorigenic. As a LOXL2 negative control, breast ErbB2+ cells were analysed (Moreno-Bueno et al., 2011).

Biology Open

Table S1. Antibodies used in Western blot, immunofluorescence and immunoprecipitation assays.

Antibody	Host	Application			Source
		WB	IF	IP	
**Primary antibodies**					
E-cadherin (ECCD-2)	Rat	1:1000	1:100		M. Takeihi, Ricken Center, Japan
N-cadherin (3B9)	Mouse	1:500			Zymed Laboratories
fibronectin	Rabbit	1:1000			Chemicon International
vimentin (V9)	Mouse	1:1000	1:100		Dako
α-vinculin	Mouse		1:100		Sigma–Aldrich
ZO-1	Rabbit		1:100		Zymed
LOXL2	Rabbit	1:1000	1:100	1:200	K. Csiszar (Hollosi et al., 2009)
Snail1	Mouse	1:1000	1:100		Cell Signaling
Flag (M2)	Mouse	1:1000	1:100		Sigma–Aldrich
HA	Rat	1:1000	1:100		Roche
pFAK	Rabbit	1:500	1:100		Cell Signaling
FAK	Mouse	1:1000			BD Transduction
pSrc	Rabbit	1:1000	1:100		Abcam
Src	Rabbit	1:1000			Cell Signaling
Histone H3	Rabbit		1:100		Santa Cruz
α-tubulin (DM1A)	Mouse	1:10,000			Sigma–Aldrich
**Secondary antibodies**					
rabbit-HRP	Donkey	1:8000			GE Healthcare
mouse-HRP	Sheep	1:8000			GE Healthcare
rat-HRP	Goat	1:8000			GE Healthcare
rat Alexa 488/546	Goat		1:1000		Molecular Probes
rabbit Alexa 488/546	Goat		1:1000		Molecular Probes
mouse Alexa 488/546	Goat		1:1000		Molecular Probes

HRP = horseradish peroxidase; WB = Western blot; IF = Immunofluorescence; IP = Immunoprecipitation.
